# Management of Metformin-Associated Lactic Acidosis by Continuous Renal Replacement Therapy

**DOI:** 10.1371/journal.pone.0023200

**Published:** 2011-08-11

**Authors:** Geoffray Keller, Martin Cour, Romain Hernu, Julien Illinger, Dominique Robert, Laurent Argaud

**Affiliations:** 1 Hospices Civils de Lyon, Groupement Hospitalier Edouard Herriot, Service de Réanimation Médicale, Lyon, France; 2 Université de Lyon, Université Lyon 1, Faculté de médecine Lyon-Est, Lyon, France; Oregon Health and Science University, United States of America

## Abstract

**Background:**

Metformin-associated lactic acidosis (MALA) is a severe metabolic failure with high related mortality. Although its use is controversial, intermittent hemodialysis is reported to be the most frequently used treatment in conjunction with nonspecific supportive measures. Our aim was to report the evolution and outcome of cases managed by continuous renal replacement therapy (CRRT).

**Methodology and Principal Findings:**

Over a 3-year period, we retrospectively identified patients admitted to the intensive care unit for severe lactic acidosis caused by metformin. We included patients in our study who were treated with CRRT because of shock. We describe their clinical and biological features at admission and during renal support, as well as their evolution. We enrolled six patients with severe lactic acidosis; the mean pH and mean lactate was 6.92±0.20 and 14.4±5.1 mmol/l, respectively. Patients had high illness severity scores, including the Simplified Acute Physiology Score II (SAPS II) (average score 63±12 points). Early CRRT comprised either venovenous hemofiltration (*n* = 3) or hemodiafiltration (*n* = 3) with a mean effluent flow rate of 34±6 ml/kg/h. Metabolic acidosis control and metformin elimination was rapid and there was no rebound. Outcome was favorable in all cases.

**Conclusions and Significance:**

Standard use of CRRT efficiently treated MALA in association with symptomatic organ supportive therapies.

## Introduction

Metformin is the recommended first-line treatment for overweight patients with type 2 diabetes mellitus [1]. The incidence of metformin-associated lactic acidosis (MALA) is rare, estimated at 2–9 patients per 100,000 patients receiving metformin per year; MALA accounts for approximately 1% of total patients admitted to intensive care units (ICU) [2]. This life-threatening complication is usually associated with a mortality rate of 30%–50% [2], [3]. The etiology of lactic acidosis is multifactorial and uncertain. Briefly, metformin increases the redox potential from aerobic to anaerobic metabolism, and inhibits gluconeogenesis by reducing hepatic lactate reuptake [4]. This situation is worsened by circulatory failure and altered tissue perfusion, both of which increase lactate production.

The optimal treatment modality for MALA is controversial and relies on nonspecific supportive measures. The use of intermittent hemodialysis may be protective, and it is recommended by many intensivists [2], [5], [6]. Despite potential advantages, continuous renal replacement therapy (CRRT) to treat MALA is poorly documented with only a few case reports available, and it has only been considered as a rescue therapy under exceptional circumstances [7]–[9]. We report on six cases of severe MALA that were successfully managed with CRRT and discuss the safety and effectiveness of CRRT when it is used for this purpose.

## Materials and Methods

The ethics committee of the Hospices Civils de Lyon approved this retrospective noninterventional study. This institutional review board waived the need for consent given the retrospective design of the project. The study was performed in compliance with the ethical standard of the Helsinki Declaration and according to the French laws.

From November 2005 to October 2008, we identified all of the patients who were admitted to our ICU for severe MALA and treated with CRRT because of hemodynamic instability. Patients were included if they met the two following criteria: blood pH<7.20 and arterial lactate >5 mmol/l [10]. An abdominal ultrasound exploration and/or a computerized tomography scanner was systematically performed to eliminate mesenteric infarction.

The following clinical features were collected at admission: age, sex, MacCabe and Knaus scores (used to assess the severity of comorbidities and functional status, respectively), Charson index (to evaluate comorbidity), preexisting chronic renal failure, use of medication potentially responsible for renal impairment, daily dose of metformin, Simplified Acute Physiology Score II (SAPS II), hemodynamics, body temperature, Glasgow coma score, number of organ failures according to Fagon *et al.*, Sequential-related Organ Failure Assessment (SOFA score), type and intensity of renal support, mechanical ventilation and vasopressor requirement, and ICU length of stay and outcome [11]–[17]. Biological data recorded at admission were pH and lactate, bicarbonate, partial oxygen pressure (PaO_2_) and partial carbon dioxide pressure (PaCO_2_) from arterial samples, and sodium, potassium, aminotransferase ALAT, creatine kinase, troponin I, creatinine, urea, glucose, hemoglobin and C-reactive protein (CRP) concentrations, as well as anion gap, prothrombin index, white blood cell and platelets counts from venous samples. The presence of metformin was identified, and both plasmatic and erythrocyte concentrations were quantified in a venous blood sample by high performance liquid chromatography (HPLC).

CRRT (i.e., continuous venovenous hemofiltration [CVVH] or hemodiafiltration [CVVHDF]) was performed with a Prismaflex device (Hospal, Meyzieu, France). We used polysulfone hollow-fiber hemofilters with a surface area of 1.2 m^2^. Blood flow rate was maintained between 150 and 250 ml/min according to the targeted ultrafiltration rate. Bicarbonate-based replacement solutions to maintain fluid balance were infused so that the predilution was equal to 30%. Anticoagulation was performed with 5000 IU unfractionated heparin, added to the priming solution and followed by a continuous infusion, with a targeted systemic activated partial thromboplastin (aPTT) at 1.5 time control. CRRT was discontinued as soon as clinical condition and renal function had improved.

Data are expressed as mean values and standard deviation (SD). Comparisons between time-based measurements were performed with two-way ANOVA with repeated measures on one factor using GraphPad Prism 5 (GraphPad Software, La Jolla, CA, USA). Statistical significance was defined at a value of *p*<0.05.

## Results

Patient clinical characteristics at admission are shown in both Table 1 and Table 2. There was one man and five women. In all cases, acute renal failure was present and associated with clinically and biologically profound extracellular dehydration. Patients 3 and 4 had previous chronic renal insufficiency without a requirement for renal replacement therapy. Except for patient 3, all of the patients took at least one of the following potentially nephrotoxic medications: diuretics, angiotensin converter enzyme inhibitors, nonsteroidal anti-inflammatory drugs, or aspirin (Table 1).

**Table 1:** Patient characteristics.

	Case 1	Case 2	Case 3	Case 4	Case 5	Case 6	Mean ± SD
**Demographics**							
Age (years)	81	81	72	54	63	64	69±11
Gender	F	F	F	M	F	F	-
**Coexisting medical conditions**							
McCabe scale	0	0	0	1	0	0	-
Knaus score	B	B	B	C	D	A	-
Charlson comorbidity index	3	5	1	2	2	1	2.3±1.5
Chronic renal failure	No	No	Yes	Yes	No	No	-
**Nephrotoxic drugs**							
Diuretics	No	Yes	No	Yes	Yes	Yes	-
Angiotensin converter inhibitors	Yes	No	No	Yes	No	Yes	-
Nonsteroidal anti-inflammators	No	No	No	No	No	No	-
Aspirin	No	No	No	Yes	No	Yes	-
**Metformin**							
Daily dose (mg)	2250	3000	1700	1000	3000	3000	2375±842

F, female; M, male; McCabe scale (life expectancy), 0: none or nonfatal underlying disease, 1: ultimately fatal disease (death≤5 years), 2: rapidly fatal disease (death≤1 year); Knaus score (functional status), A: no daily activity limitation, the patient was in good health, B: moderate limitation of activity because of a chronic medical problem, C: strong limitation of activity due to disease, D: severe limitation and/or restriction of activity due to disease; Charlson comorbidity index, components (weights): myocardial infarct (1), congestive heart failure (1), peripheral vascular disease (1), cerebrovascular disease (1), dementia (1), chronic pulmonary disease (1), connective tissue disease (1), ulcer disease (1), mild liver disease (1), diabetes (1), hemiplegia (2), moderate or severe renal disease (2), diabetes with end-organ damage (2), any tumor (2), leukemia (2), lymphoma (2), moderate or severe liver disease (3), metastatic solid tumor (6), AIDS (6).

**Table 2:** Illness severity.

	Case 1	Case 2	Case 3	Case 4	Case 5	Case 6	Mean ± SD
**Admission vitals**							
Heart rate (beats/min)	104	101	83	82	78	120	95±16
Mean arterial blood pressure (mmHg)	44	41	48	38	55	54	47±7
Respiratory rate (breaths/min)	32	20	30	30	29	40	30±7
Body temperature (°C)	33.0	34.5	33.0	35.8	35.9	30.9	33.9±1.9
Diuresis (ml/h)	4	9	0	88	8	0	18±34
Glasgow coma score	15	11	8	15	11	12	12±3
**Multiple organ failure**							
Cardiovascular dysfunction	Yes	Yes	Yes	Yes	Yes	Yes	-
Renal dysfunction	Yes	Yes	Yes	Yes	Yes	Yes	-
Respiratory dysfunction	No	Yes	No	Yes	Yes	Yes	-
Neurological dysfunction	No	Yes	Yes	No	No	No	-
Hepatic dysfunction	No	No	Yes	No	No	No	-
Hematological dysfunction	No	No	No	No	No	No	-
Number of organ dysfunctions	2	4	4	3	3	3	3.2±0.8
SOFA score	9	14	15	8	12	14	12±3
**Symptomatic intensive therapies**							
Renal replacement therapy (days)	7	12	15	2	3	5	7±5
Mechanical ventilation (days)	0	19	0	5	15	9	8±8
Vasoactive drugs (days)	3	9	4	1	2	5	4±3
Norepinephrine (µg/kg/min)	0.3	1.3	0.8	0.2	0.2	0.9	0.6±0.5
**SAPS II**	66	74	73	51	46	66	63±12

SOFA, Sequential-related Organ Failure Assessment; SAPS II, Simplified Acute Physiology Score II.

All of the patients presented with clinical nonspecific symptoms such as malaise, myalgia, drowsiness, or abdominal pain, as well as hemodynamic failure which required fluid challenge and vasopressive support (Table 2). Hypothermia was systematically present; four patients required mechanical ventilation (Table 2).

Biological characteristics at admission are presented in Table 3. None of the patients experienced severe hypoglycemia (Table 3). Inflammatory syndrome, if present, was minor since CRP level was always below 30 mg/l.

**Table 3:** Biological data at admission.

	Case 1	Case 2	Case 3	Case 4	Case 5	Case 6	Mean ± SD
**Arterial blood gases**							
Arterial pH	7.04	6.81	6.71	7.09	7.15	6.72	6.92±0.20
Arterial lactate (mmol/l)	16.2	19.1	15.6	9.8	6.6	19.0	14.4±5.1
Bicarbonate (mmol/l)	5	2	1	7	15	1	5±5
Anion gap (mmol/l)	51	42	51	52	36	54	48±7
PaCO_2_ (mmHg)	16.5	15.0	24.8	23.3	43.5	9.0	21.8±12.0
PaO_2_/FiO_2_ (mmHg)	250	238	194	181	233	313	235±47
**Standard biochemistry**							
Sodium (mmol/l)	140	135	131	139	136	133	136±3
Potassium (mmol/l)	5.0	6.2	7.5	6.7	7.6	7.2	6.7±1.0
Glycemia (mmol/l)	12.5	2.2	9.3	13.0	8.7	6.3	8.7±4.0
Urea (mmol/l)	26	26	39	24	21	31	28±7
Creatinine (µmol/l)	670	416	841	585	372	723	601±181
Aminotransferase ALAT (IU/l)	12	29	128	40	13	15	39±45
Creatine kinase (IU/l)	150	63	554	124	43	484	236±224
Troponin I (ng/ml)	0.18	<0.1	1.33	<0.1	<0.1	1.14	0.9±0.6
**Hematology**							
Hemoglobin (g/l)	81	116	93	100	128	86	101±18
Platelets (10^9^/l)	160	161	350	251	363	401	281±106
White blood cell (10^9^/l)	18	25	27	17	13	39	23±9
Prothrombin index (%)	50	48	24	60	41	28	42±14
**Metformin concentration**							
Plasma (mg/l), N<1 mg/l	80.0	125	74.4	36.4	54.9	61.9	72.1±30.1
Erythrocyte (mg/l), N<0.81 mg/l	25.8	26.8	22.5	14.7	51.8	20.6	27.0±12.9

N, laboratory level limit; PaCO_2_, partial carbon dioxide pressure in arterial blood; PaO_2_, partial oxygen pressure in arterial blood; FiO_2_, inspiratory fraction of oxygen.

**Figure 1:**
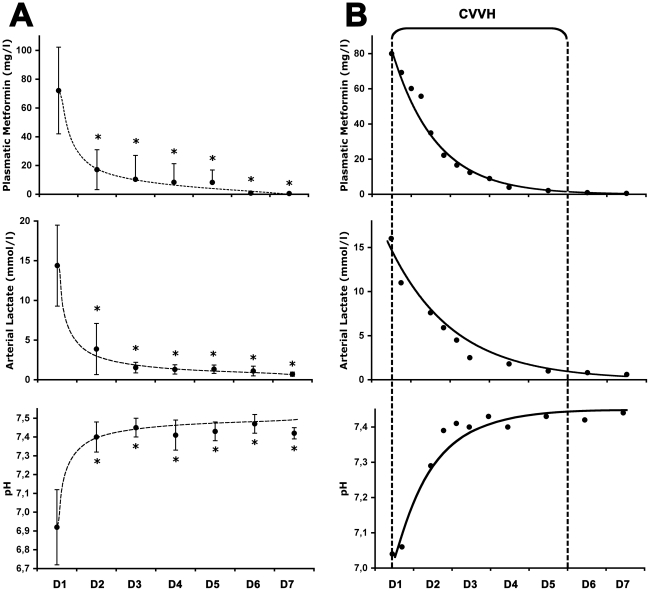
Acidosis, lactate and metformin levels under continuous renal replacement therapy. Panel A: Data from all patients, expressed as mean ± SD, showing that metabolic acidosis, as well as the excessive dose of metformin observed at admission (day 1, D1), were dramatically reduced from day 2 (D2). * p<0.01 versus D1. Panel B: Typical evolution in case patient 1 of both metformin plasma concentrations and metabolic disorders, which were controlled within 2 days of initiating continuous venovenous hemofiltration (CVVH), i.e. without dialysate.

Continuous renal replacement management, as well as outcomes, are shown in Table 4. CVVH and CVVHDF were each used in three patients. Metabolic acidosis, as well as metformin plasma concentrations, were dramatically reduced in the first 24 h and/or normalized on the second day in every case (Figure 1). There was no rebound in acidosis. The mean individual rates of metformin elimination, from the blood compartment, in the first 24 hours after admission was estimated from 1.5 to 4.0%/h (Table 4). CRRT was well tolerated in our patients. There was no occurrence of CRRT-associated complications such as bleeding. Renal replacement was not necessary after discharge for any of the patients, and kidney function recovered prior levels in each case. All of the patients were transferred to a medical ward before they were discharged to their homes.

**Table 4:** Continuous renal replacement therapy and outcomes.

	Case 1	Case 2	Case 3	Case 4	Case 5	Case 6	Mean ± SD
**Type**							
CVVH or CVVHDF	CVVH	CVVH	CVVHDF	CVVH	CVVHDF	CVVHDF	-
**Therapy parameters**							
Blood flow rate (ml/min)	200	180	150	200	180	250	193±33
Effluent rate (ml/kg/h)	34	33	39	22	38	36	34±6
Dialysate rate (ml/h)	0	0	2500	0	2000	500	1250±1215
Replacement fluid rate (ml/h)	1600	2500	2000	2500	2500	2800	2317±436
Filtration fraction (%)	13	30	22	29	17	19	22±7
Initial fluid removal (ml/h)	0	50	0	0	100	0	25±42
Length (days)	5	12	15	1	3	5	7±5
**Metformin clearance**							
Rate of elimination (%/h)	2.7	4.0	1.5	1.6	2.1	2.3	2.4±0.9
**Outcomes**							
ICU length of stay (days)	11	26	17	8	22	9	16±7
Survival to discharge	Yes	Yes	Yes	Yes	Yes	Yes	-
Discharge at home	Yes	Yes	Yes	Yes	Yes	Yes	-

CVVH, continuous venovenous hemofiltration; CVVHDF, continuous venovenous hemodiafiltration.

## Discussion

To our knowledge, this study represents the largest case series of MALA managed by early CRRT. All six patients had a favourable outcome despite severe initial metabolic disorders associated with multiple organ failure.

MALA is strictly defined by arterial lactate >5 mmol/l and blood pH<7.35 within the context of recent metformin exposure [10], [18]. It is the most frequent pattern of lactic acidosis related to metformin use [10]. This confusing term of MALA, shared by many different nosologic entities, is caused by an acute metformin accumulation [10]. Because metformin normally undergoes rapid and unchanging glomerular filtration and tubular excretion, MALA occurs only if renal function is altered or in rare cases of massive metformin ingestion [8], [10]. Dehydration was the precipitating factor responsible for acute renal failure in our patients. In addition, the use of nephrotoxic drugs and/or chronic renal failure may have favoured the development of MALA.

Our patients presented with classical symptoms of MALA within a context that suggested metformin accumulation [2], [10]. However, the diagnosis of MALA was made only once other causes of lactic acidosis (e.g., mesenteric infarction or septic shock) were excluded. Plasma metformin concentrations, ideally measured in the emergency room, helped us to ensure the correct diagnosis. These measurements are usually performed to eliminate metformin as the cause of lactic acidosis in patients with low plasma levels. However, the concentration of metformin in erythrocytes may be more useful, since it better reflects tissue accumulation [19]. Thus, as reported in this case series of metformin-treated patients, severe lactic acidosis associated with acute renal failure and/or other sepsis-like symptoms should systematically lead physicians to request metformin assays. Using this restrictive approach, we were able to generate a report on a rare, albeit small, series of MALA-only patients.

There are some concerns about using renal replacement therapy to manage MALA. For example, it is not certain whether rapid metformin elimination, either by intermittent hemodialysis or CRRT, is an appropriate endpoint in studies of MALA therapy [10]. Indeed, as previously reported by Lalau and Race, metformin (and also lactate) concentrations are not closely associated with prognosis [20]. In addition, increased levels of both metformin and lactate could even have beneficial cardiovascular, metabolic, and cytoprotective properties [21], [22]. With regard to lactate management, it is now well established that lactate is not an acidogenic substance; the amount removed by replacement therapy with dialysis using bicarbonate-buffered fluids is negligible when compared to the overall plasma lactate clearance [23]. In the same way, the CRRT that was used in this case series is considered by some authors to be a supportive measure only to buffer metabolic acidosis and control volemia [10].

Even if there is also no consensus as to the best replacement therapy, hemodialysis appears to be the first-line treatment in association with symptomatic organ failure treatment [2], [4]–[6], [24]. In contrast, CRRT to manage MALA has only received attention in a few case reports [7]–[9], [25], [26]. Surprisingly, it usually appears to have been used as a rescue therapy either when high flow rates are set or in combination with hemodialysis [8], [25], [26]. In our practice, we began CRRT as early as possible after patients were admitted to the ICU. Using unfractionated heparin as anticoagulation, we did observe in the present study any of the classical CRRT-associated complications such as bleeding or extracorporeal circuit clotting [27]. We chose to use of CVVHDF, rather than CVVH, in cases of severe and threatening hyperkalemia. We used also a flow rate of the total effluent (the sum of the dialysate and ultrafiltrate) averaging 34±6 ml/kg/h; i.e., a “standard” dose of replacement solution when compared to the very high flow rates (50 to 80 ml/kg/h) sometimes proposed to treat MALA [25], [26]. As classically reported, renal recovery (urine output increase and spontaneous urea/creatinine decrease), metabolic state improvement, fluid overload correction, as well as hemodynamic stability were the mean criteria to decide cessation of CRRT [28].

CRRT seems more physiologically appropriate than intermittent hemodialysis in this setting for several reasons. First, because of the drug's low molecular weight and lack of protein binding, conventional modalities of treatment (i.e., dialysis and/or ultrafiltration) can perform high plasma clearance of metformin [24]. Second, metformin has a large volume of distribution (3.1 l/kg) secondary to intracellular penetration [29]. Seidowsky *et al.* determined recently that 15 cumulative hours of hemodialysis were needed to return patients to therapeutic levels of metformin [5]. CRRT can be used for extended durations and maximizes metformin removal, with a rate of metformin elimination from the blood compartment averaging 2.4±0.9%/h. If prolonged renal therapy is required, initiation of CRRT upon patient admission may be a fast and convenient treatment. Third, all of our patients had circulatory failure upon admission. Because of this, we needed to avoid the detrimental impact of highly intermittent dialysis on hemodynamics, which is caused by major variations in solutes, bicarbonate, electrolytes, pH, and volemia. Thus, CRRT may be a superior choice for MALA, because it gradually removes solutes and places patients in a prolonged physiologically steady state [30].

The reported mortality in patients with MALA was initially very high, nearing 50% [3], [18]. More recently, Peters *et al.* reported a 30% death rate in patients admitted to the ICU with MALA [2]. Awareness of metformin complications, as well as better organ failure treatment in ICUs may be the reasons for this decrease in mortality. Indeed, symptomatic management of organ failure (e.g., mechanical ventilation and/or vasoactive drugs) at admission was our priority even before ensuring diagnosis and starting CRRT as a specific treatment. Furthermore, in the present study, in addition to CRRT, we aggressively corrected both the precipitating and the underlying conditions of metformin accumulation by nonspecific therapeutic measures, which could also explain the favourable outcomes we observed.

In summary, early CRRT appears to be a safe and effective means of managing MALA in patients with hemodynamic instability and its use should become more widespread. This modality of replacement therapy, in conjunction with other symptomatic intensive therapies, rapidly corrects metabolic disorders and efficiently eliminates metformin when the standard guidelines for use are followed. However, further studies are needed to determine the most adequate ways of administering this CRRT in patients with MALA.
